# Relationship between serum triglyceride to high-density lipoprotein cholesterol ratio and sarcopenia occurrence rate in community-dwelling Chinese adults

**DOI:** 10.1186/s12944-020-01422-4

**Published:** 2020-12-04

**Authors:** Na Wang, Mengjun Chen, Danhong Fang

**Affiliations:** 1 Health Care Center, the First Affiliated Hospital of Wenzhou Medical University, Wenzhou, 325002 Zhejiang China; 2 Gastroenterology Department, the First Affiliated Hospital of Wenzhou Medical University, Wenzhou, 325002 Zhejiang China; 3 Cardiology Department, the First Affiliated Hospital of Wenzhou Medical University, Nan Bai Xiang Street, Ouhai District, Wenzhou, 325002 Zhejiang China

**Keywords:** Sarcopenia, Triglyceride, High-density lipoprotein cholesterol, Community-dwelling Chinese adults

## Abstract

**Background:**

A study conducted on elderly Korean men showed that a high serum triglyceride to high-density lipoprotein cholesterol (TG/HDL-C) ratio was associated with a high risk of developing sarcopenia. We aimed to determine such an association in community-dwelling Chinese adults.

**Methods:**

From May 2016 to August 2017, we conducted a cross-sectional study on Chinese adults at the First Affiliated Hospital of Wenzhou Medical University. Univariate and multivariate logistic regression analyses were applied to evaluate a possible relationship between TG/HDL-C ratio and sarcopenia occurrence.

**Results:**

We included 2613 adults in this study, with 13.85% presenting with sarcopenia. The odds ratios (ORs) for TG and HDL-C were 0.67 (95% confidence interval [CI]: 0.51–0.87), and 1.97 (95% CI: 1.49–2.61), respectively. Moreover, TG/HDL-C ratio was independently associated with sarcopenia status (OR: 0.63; 95% CI: 0.49–0.81).

**Conclusions:**

We found that TG and HDL-C were, respectively, negatively and positively associated with sarcopenia occurrence rate in community-dwelling Chinese adults. However, a negative association was found between sarcopenia occurrence rate and TG/HDL-C ratio.

**Supplementary Information:**

The online version contains supplementary material available at 10.1186/s12944-020-01422-4.

## Introduction

Sarcopenia, which was defined by the 2014 Asian Working Group for Sarcopenia (AWGS) as the age-related reduction of muscle mass and function, has drawn public attention worldwide [1–5]. In 2019, however, the AWGS regarded possible sarcopenia as either reduced muscle strength or reduced physical ability. The global prevalence of sarcopenia, which has gradually become a public health problem, has been on the rise, and its incidence is at 5.5–25.7% [6–8].

Presently, the pathophysiology of sarcopenia remains unelucidated. Many variables, such as age, inflammation, hormonal changes, and cachexia are considered as risk factors of sarcopenia [9–15].

Serum lipid profile is commonly tested in a clinical setting. Jaekyung No et al. recently published a meta-analysis and confirmed that serum triglycerides (TG) and high-density lipoprotein cholesterol (HDL-C) levels had a positive and negative correlation with sarcopenia, respectively [16]. Tae-Ha et al. demonstrated that a higher TG/HDL-C ratio was associated with an increased rate of sarcopenia occurrence in elderly Korean men [17]. Therefore, we aimed to determine a potential association between serum lipid profile and sarcopenia in community-dwelling Chinese adults.

## Methods

### Study population

From May 2016 to August 2017, we conducted a cross-sectional study on individuals undergoing routine health follow-up at the First Affiliated Hospital of Wenzhou Medical University. We included participants aged ≥18 years who underwent serum lipid profile assay and bioelectrical impedance analysis (BIA) during the study period. We excluded individuals aged < 18 years; those taking anti-dyslipidemic medications; and those with a history of stroke, malignant tumor, chronic kidney disease, liver disease, or thyroid disease.

The study was approved by the hospital’s Institutional Review Board (IRB). Given its cross-sectional nature, the need for participant informed consent was waived by the IRB as confidentiality was assured. The study was conducted in in accordance with the principles of the Declaration of Helsinki.

### Data collection

With the use of predesigned questionnaires, we collected the following patient data: mode of life and comorbidities, as well as the results of BIA, blood analysis, and biochemical and anthropometric measurements.

Smoking and alcohol consumption: Alcohol consumptions > 70 g and 140 g per week for women and men, respectively, were considered as heavy drinking. Smoking status was divided into the following 3 categories: current smoker (has been smoking for at least 6 months, or abandoned smoking < 2 years ago), past smoker (stopped smoking at least 2 years ago), and non-smoker (never smoked).

Comorbidities included diabetes mellitus (DM), hypertension (HTN), and hyperuricemia. DM was define as random plasma glucose (PG), fasting plasma glucose (FPG), or 2-h PG ≥ 200 mg/dL, ≥ 126 mg/dL, or ≥ 200 mg/dL, respectively [18]. Systolic blood pressure (SBP) and diastolic blood pressure (DBP) were measured for all participants in the morning during medical examination. HTN was defined as SBP ≥140 mmHg or DBP ≥90 mmHg [19]. Hyperuricemia was defined as serum uric acid (UA) levels > 6 mg/dL and > 7.0 mg/dL in females and males, respectively [20]. Additionally, we obtained comorbidity and drug histories from self-reports.

BIA (InBody770; InBody Japan Inc., Tokyo, Japan) was used to monitor patient appendicular skeletal muscle mass (ASM, in kg). Thereafter, skeletal muscle mass index (SMI, kg/m^2^) was computed using the formula, SMI = ASM (kg)/ height^2^ (m^2^). Moreover, according to the AWGS 2019 Consensus [1], sarcopenia was diagnosed in males and females with SMI < 7.0 kg/m^2^ and < 5.7 kg/m^2^, respectively. Participants with body mass index (BMI) > 25 kg/m^2^ were considered to be overweight.

The following blood parameters were measured on the morning of the day when the medical examination was performed: TG, HDL-C, low-density lipoprotein cholesterol (LDL-C), total cholesterol (TC), glycated hemoglobin (HbA1c), FPG, albumin, UA, hemoglobin (Hb), white blood cell count (WBC), and platelet count (PLT).

### Statistical analysis

Continuous and categorical variables were presented as medians (ranges) and frequencies (percentages), respectively. We used receiver operating characteristic (ROC) curves to assess the diagnostic accuracy of lipid profile for sarcopenia. The optimal indicator was selected based on the area under the curve (AUC) and Youden index. Participants were grouped, according to the selected indicator, in the high and low groups. Differences in continuous and categorical variables were compared with the Mann-Whitney and χ2 tests, respectively. Univariate and multivariate logistic regression models were used to evaluate the effects of the different factors on the risk of sarcopenia occurrence; moreover, TG, HDL-C, and TG/HDL-C ratio were used as separate logistic regression models. We performed backward stepwise selection using the Akaike information criterion (AIC) to identify variables for multivariate logistic regression models. Interaction terms were added to the multivariate logistic regression analysis in order to get rid of confounding factors. In addition, individuals were stratified by quartiles, and the χ^2^ test was used to compare the different sarcopenia occurrence rates.

Statistical analyses were performed using R version 3.6.1 (https://www.r-project.org/). All analyses were two-sided, and a *P* value < 0.05 was considered statistically significant.

## Results

### Participant characteristics

We enrolled 2613 participants, and the medians of ASM and SMI were 20.14 (8.9–32.98) kg and 7.27 (4.35–11.50) kg/m^2^, respectively. The medians of TG and HDL-C were 127.44 (35.40–1896.56) mg/dL and 45.23 (17.40–108.25) mg/dL, respectively. Other clinical features are shown in Table 1. Of 2613 participants, 362 (13.85%) were diagnosed with sarcopenia.

**Table 1:** Clinical features according to the ratio of TG/HDL-C

Variables	Total (*N* = 2613)	TG/HDL-C ratio		*P* value
		Low (*N* = 1266)	High (*N* = 1347)	
Age (years)	48 (18–91)	47 (18–91)	49 (21–83)	< 0.01
Gender(%)				
Male	1614 (61.80)	564 (44.50)	1050 (78.00)	< 0.01
Female	999 (38.20)	702 (55.50)	297 (22.00)	
BMI (kg/m2)	23.81 (15.05–43.02)	22.41 (15.05–32.63)	24.92 (16.44–43.02)	< 0.01
Overweight(%)	886 (33.90)	240 (19.00)	646 (48.00)	< 0.01
ASM (kg)	20.14 (8.90–32.98)	17.69 (8.90–28.73)	21.56 (11.67–32.98)	< 0.01
SMI (kg/m^2^)	7.27 (4.35–11.50)	6.70 (4.35–9.38)	7.64 (4.99–11.50)	< 0.01
Systolic blood pressure (mmHg)	124 (77–196)	121 (77–194)	126 (86–196)	< 0.01
Diastolic blood pressure (mmHg)	74 (40–119)	71 (40–111)	76 (41–119)	< 0.01
Blood and biochemical parameters				
Fasting plasma glucose (mg/dL)	86.40 (54.00–327.60)	84.60 (54.00–246.60)	88.20 (55.80–327.60)	< 0.01
Glycated hemoglobin(%)	5.40 (3.30–16.70)	5.30 (3.90–11.90)	5.50 (3.30–16.70)	< 0.01
Total cholesterol (mg/dL)	199.87 (85.83–402.84)	194.07 (85.83–400.52)	206.44 (103.22–402.84)	< 0.01
LDL cholesterol (mg/dL)	119.85 (30.54–301.16)	115.01 (30.54–301.16)	124.49 (42.53–272.94)	< 0.01
HDL cholesterol (mg/dL)	45.23 (17.40–108.25)	53.74 (25.90–108.25)	39.43 (17.40–74.23)	< 0.01
Triglycerides (mg/dL)	127.44 (35.40–1896.56)	88.50 (35.40–247.80)	192.93 (82.31–1896.56)	< 0.01
Uric acid (μmol/L)	5.76 (1.73–12.89)	5.09 (1.73–11.51)	6.375 (2.81–12.89)	< 0.01
Albumin(g/dL)	4.44 (2.97–5.60)	4.43 (3.32–5.60)	4.45 (2.97–5.48)	< 0.01
Leukocyte count (×10^9/L)	5.88 (1.74–14.72)	5.44 (1.74–14.33)	6.25 (2.90–14.72)	< 0.01
Hemoglobin(g/dL)	146 (42–191)	139 (42–181)	151 (77–191)	< 0.01
Platelet(×10^9/L)	229 (31–638)	230 (31–638)	227 (90–489)	0.194
Clinical parameters				
Smoking(%)				
Never	1922 (73.6)	1052 (83.1)	870 (64.6)	< 0.01
Past	60 (2.3)	21 (1.7)	39 (2.9)	0.04
Current	631 (24.1)	193 (15.2)	438 (32.5)	< 0.01
Heavy drink(%)	361 (13.8)	121 (9.6)	240 (17.8)	< 0.01
Diabetes mellitus (%)	257 (9.8)	61 (4.8)	196 (14.6)	< 0.01
Hypertension(%)	726 (27.8)	292 (23.1)	434 (32.2)	< 0.01

*BMI* Body mass index, *ASM* Appendicular skeletal muscle mass, *SMI* Skeletal muscle mass index

### Cutoffs and clinical features according to grouping

By ROC curves, TG, diagnostic performances of lipid profile were evaluated (Supplementary Figure). The cutoff for TG was 138.50 mg/dL with an AUC of 0.63 and a Youden Index of 0.2 (sensitivity: 0.72; specificity: 0.48; *P* < 0.01). The cutoff for HDL-C was 42.72 mg/dL with an AUC of 0.64 and a Youden Index of 0.22 (sensitivity: 0.78; specificity: 0.44; *P* < 0.01). The cutoff for TG/HDL-C ratio was 2.78 with an AUC of 0.64 and a Youden Index of 0.22 (sensitivity: 0.67; specificity: 0.55; *P* < 0.01). Based on the Youden index and AUC, TG/HDL-C ratio was selected as the optimal indicator. Participants were classified into TG/HDL-C^low^ group (TG/HDL-C ratio < 2.78) and TG/HDL-C^high^ group (TG/HDL-C ratio ≥ 2.78).

There were 1266 and 1347 individuals in the low and high groups, respectively. We diagnosed 243 (19.19%) and 119 (8.83%) individuals in the low and high groups, respectively, with sarcopenia. The clinical features are displayed in Table 1. Compared to participants in the low group, those in the high group showed higher ASM (*p* < 0.01), SMI (*p* < 0.01), and BMI (*p* < 0.01), were predominantly elderly (*p* < 0.01), and were and males (*p* < 0.01).

### Univariate and multivariate logistic regression analyses

Univariate and multivariate analyses were used to assess the possible effects of clinical variables on sarcopenia. As depicted in Table 2, factors such as the ratio of TG/HDL-C (high vs. low, odds ratio [OR]: 0.63, 95% confidence interval [CI]: 0.49–0.81), overweight status (yes vs. no, OR: 0.04, 95% CI: 0.04–0.07), and age (> 65 vs. ≤65, OR: 2.10, 95% CI: 1.43–3.10) kept independent effects on sarcopenia, with C-index of 0.73. Separate results for TG and HDL-C are displayed in Table 3. TG (> 138.50 vs. ≤138.50, OR: 0.67, 95% CI: 0.51–0.87) and HDL-C (> 42.72 vs. ≤42.72, OR: 1.97, 95% CI: 1.49–2.61) were also independently associated with sarcopenia occurrence.

**Table 2:** Univariate and multivariate analyses for sarcopenia

Variables	Univariate analysis		Multivariate analysis	
	Crode OR (95% CI)	*P* value	Adjust OR (95% CI)	*P* value
Age, > 65 vs. ≤65	1.89 (1.32–2.70)	< 0.01	2.10 (1.43–3.10)	< 0.01
Gender, male vs. female	1.78 (1.42–2,22)	< 0.01	1.16 (0.90–1.49)	0.26
Smoking (%)				
Past vs. never	0.78 (0.35–1.74)	0.55		
Current vs. never	0.84 (0.64–1.10)	0.20		
Heavy drink, yes vs. no	0.70 (0.49–1.00)	0.05	0.95 (0.64–1.41)	0.80
Diabetes mellitus, yes vs. no	0.71 (0.47–1.07)	0.10		
Hypertension, yes vs. no	0.76 (0.58–0.98)	0.04	0.99 (0.74–1.32)	0.92
Hyperuricemia, yes vs. no	0.56 (0.42–0.76)	< 0.01	0.97 (0.70–1.35)	0.86
Overweight, yes vs. no	0.03 (0.02–0.07)	< 0.01	0.04 (0.02–0.07)	< 0.01
TG/HDL-C ratio high vs. low	0.41 (0.32–0.52)	< 0.01	0.63 (0.49–0.81)	< 0.01

**Table 3:** The separate results of multivariate analyses for TG and HDL-C

Variables	Multivariate analysis for TG		Multivariate analysis for HDL-C	
	Crode OR (95% CI)	*P* value	Adjust OR (95% CI)	*P* value
Age, > 65 vs. ≤65	2.12 (1.42–3.16)	< 0.01	2.08 (1.40–3.11)	< 0.01
Gender, male vs. female	0.83 (0.65–1.07)	0.16	0.90 (0.70–1.17)	0.44
Heavy drink, yes vs. no	1.00 (0.67–1.48)	0.99	0.89 (0.60–1.33)	0.58
Hypertension, yes vs. no	0.99 (0.74–1.32)	0.94	0.96 (0.72–1.29)	0.95
Hyperuricemia, yes vs. no	0.96 (0.69–1.34)	0.82	0.99 (0.71–1.37)	0.81
Overweight, yes vs. no	0.04 (0.02–0.08)	< 0.01	0.04 (0.02–0.07)	< 0.01
TG > 138.50 vs. ≤138.50	0.67 (0.51–0.87)	< 0.01		
HDL > 42.72 vs. ≤42.72			1.97 (1.49–2.61)	< 0.01

The TG/HDL-C ratio was an independent predictor of sarcopenia occurrence on multivariate logistic regression analysis (Table 4) (OR: 0.75, 95% CI: 0.63–0.89), with C-index of 0.75.

**Table 4:** Multivariate analysis for sarcopenia with TG/HDL-C as a continuous predictor

Variables	Adjust OR (95% CI)	*P* value
Age, > 65 vs. ≤65	2.12 (1.42–3.16)	< 0.01
Gender, male vs. female	0.86 (0.67–1.11)	0.26
Heavy drink, yes vs. no	0.98 (0.66–1.46)	0.92
Hypertension, yes vs. no	0.98 (0.73–1.31)	0.97
Hyperuricemia, yes vs. no	0.99 (0.71–1.38)	0.88
Overweight, yes vs. no	0.04 (0.02–0.08)	< 0.01
TG/HDL-C ratio high vs. low	0.75 (0.63–0.89)	< 0.01

### Association between sarcopenia occurrence rate and TG/HDL-C ratio

**Fig. 1:**
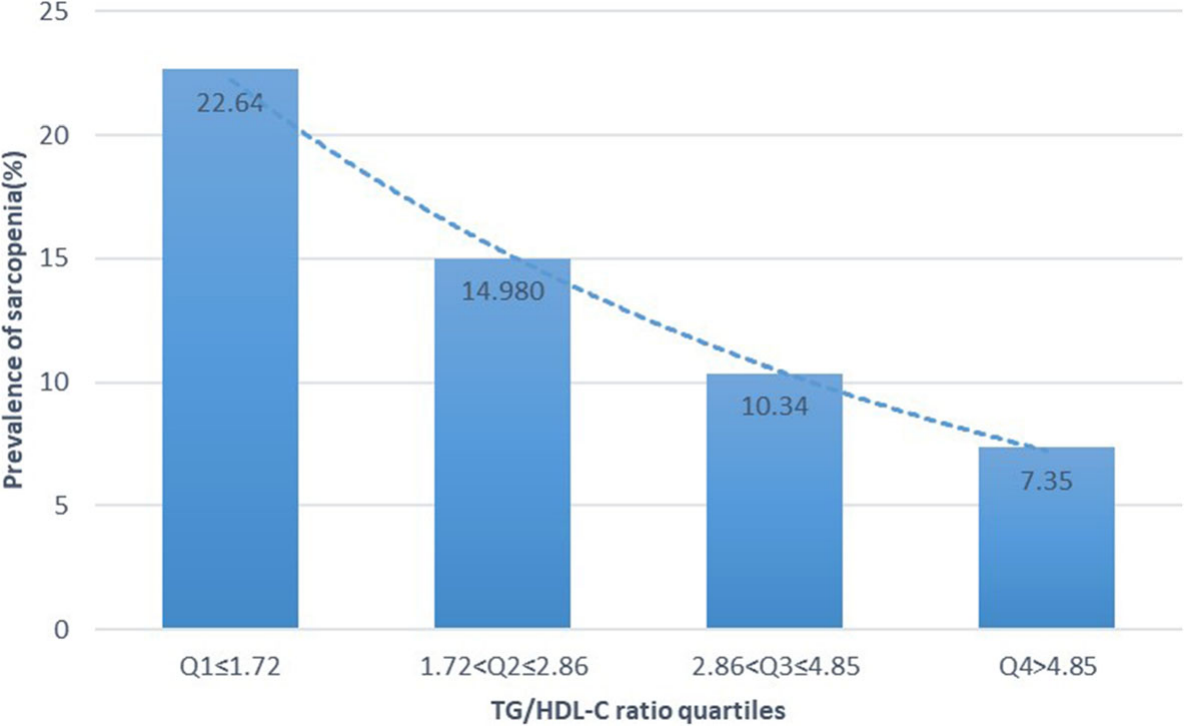
The prevalence of sarcopenia according to the TG/HDL-C ratio quartiles

Participants were stratified by quartiles as shown in Fig. 1. Sarcopenia occurrence rate reduced with increase in TG/HDL-C ratio (*p* < 0.01). Furthermore, multivariate logistic regression analysis showed that TG/HDL-C ratio quartiles were independent factors in sarcopenia occurrence. (Q2 vs. Q1: OR: 0.77, 95% CI: 0.57–1.03; Q3 vs. Q1: OR: 0.65, 95% CI: 0.46–0.92; Q4 vs. Q1: OR, 0.55, 95% CI: 0.38–0.81; Table 5). There was no significant relationship between age and TG/HDL-C ratio (OR: 1.36, 95% CI: 0.62–2.98, *p* = 0.45), though age-by-TG/HDL interaction term.

**Table 5:** Multivariate analysis for sarcopenia with TG/HDL-C by quartiles

Variables	Adjust OR (95% CI)	*P* value
Age, > 65 vs. ≤65	2.10 (1.41–3.13)	< 0.01
Gender, male vs. female	0.89 (0.69–1.15)	0.38
Heavy drink, yes vs. no	0.96 (0.64–1.42)	0.82
Hypertension, yes vs. no	0.99 (0.74–1.32)	0.94
Hyperuricemia, yes vs. no	0.98 (0.70–1.36)	0.9
Overweight, yes vs. no	0.04 (0.02–0.08)	< 0.01
TG/HDL-C ratio Q2 vs. Q1	0.77 (0.57–1.03)	0.08
Q3 vs. Q1	0.65 (0.46–0.92)	0.01
Q4 vs. Q1	0.55 (0.38–0.81)	< 0.01

## Discussion

We found a negative association between sarcopenia and TG/HDL-C ratio in community-dwelling adults in China. Tae-Ha et al. reported a positive association between TG/HDL-C ratio and sarcopenia occurrence rate in elderly Korean males; moreover, insulin resistance had a similar relationship with sarcopenia. However, their study was generally limited to elderly people with sex differences [17]. Our study was conducted on a larger number of participants of both sexes, with a median age of 48 years. Additionally, there were methodological differences between our study and theirs. The prevalence of sarcopenia based on TG/HDL-C ratio quartiles was identified by adjusting for age, cigarette smoking, alcohol intake, BMI, and physical activity in the study by Tae-Ha et al. These adjusting factors were chosen based on their clinical importance. In our study, the adjusting factors was identified by backward stepwise selection with the AIC in univariate logistic regression analysis. The selected adjusting factors included TG/HDL-C ratio, overweight status, and age. Genetic and lifestyle factors in different populations contribute to inter-individual variations in serum cholesterol and TG levels. Previous studies have identified special loci in different populations. For instance, there were novel loci near MYL2 and HECTD2 associated with HDL-C production in Korean individuals [21]. The missense variants at PNPLA3 and PKD1L3 were shown to correlate with TG and LDL-C production in a Chinese population [22]. The difference in the distribution of genetic polymorphism in Chinese and Koreans remains unillustrated; however, the difference in lipid profiles between adolescents in these countries has been demonstrated. Korean adolescents had higher levels of TC, LDL-C, TC/HDL-C, and LDL-C/HDL-C compared to Korean-Chinese adolescents [18]. Additionally, lifestyle factors, such as alcohol abuse, dietary habits, and physical activity, may affect lipid profiles [23].

There was a negative and positive association, respectively, between sarcopenia occurrence rate and TG and HDL-C levels. As with TG levels, the TG/HDL-C ratio had a negative association with sarcopenia occurrence rate. However, this demonstrated that the association between TG/HDL ratio and sarcopenia occurrence cannot be adequately evaluated if one of its components is positively while the other is negatively associated with sarcopenia occurrence rate.

The lipid profile, a widely used test, is cost-effective and has easy performance procedures. Among the different lipid profile parameters, it was found that medium-chain TG could be a feasible nutrient for sarcopenia, as medium-chain TG could enhance muscle strength by increasing ghrelin levels [24]. Moreover, Stella et al. suggested that muscle loss could be attenuated by plasma TG, which was generated from omega 3 fatty acids [25]. We found that higher HDL-C levels were associated with increased sarcopenia occurrence rates, akin to the findings of prior studies [26–28]. For example, Caitlin et al. [26] suggested that children with lower grip strength had higher HDL-C levels. According to these findings, an association between sarcopenia occurrence risk and TG or HDL-C levels should be considered. Thus, an appropriate supplementation of fatty foods could help in building muscle function; however, further studies are needed to determine the degree of fatty food supplementation required.

### Study strengths and limitations

This was a large cross-sectional study conducted on 2613 Chinese individuals that provided new and contrary findings compared to those reported by a previous Korean study [17]. Our results indicated that the relationship between lipid profile and sarcopenia occurrence may explained by different nationalities and territories.

However, there were some limitations. First, due to the cross-sectional nature of the study, causality could not be determined, and there is a possibility of reverse causality bias. Additionally, the study was not registered in a database of clinical studies. Second, due to the lack of data, the new sarcopenia definition was not taken into consideration; this definition suggested the diagnosis of sarcopenia based on 2 elements: low muscle mass and function [1]. Third, many of the individuals involved in this study were aged < 65 years and without any severe disease. Thus, our results may not apply to older and critically ill patients. Finally, the cutoff was determined by the ROC curve, with restrictive sensitivity and specificity.

## Conclusion

Generally, a low TG/HDL-C ratio was a potential indicator for sarcopenia occurrence regardless of whether it is a continuous variable or a categorical variable. In clinical practice, TG supplementation and HDL-C control should be implemented to reduce the impact and rate of sarcopenia occurrence, especially in Chinese patients.

Future prospective studies with more comprehensive data and larger sample sizes are required to validate the relationship lipid profile and sarcopenia occurrence.

## Supplementary Information

**Additional file 1: Supplementary Figure.** Receiver operating characteristic (ROC) curve analyses of serum TG, HDL-C and TG/HDL-C ratio in sarcopenia status.

## Data Availability

The datasets used and analyzed during the current study will be provided by the corresponding author upon reasonable request.
