# Supplementary figures and images for: Relationship between serum triglyceride to high-density lipoprotein cholesterol ratio and sarcopenia occurrence rate in community-dwelling Chinese adults
Source: Lipids Health Dis. 2020 Dec 4;19:248. doi: 10.1186/s12944-020-01422-4 (PMC7716486; doi:10.1186/s12944-020-01422-4)

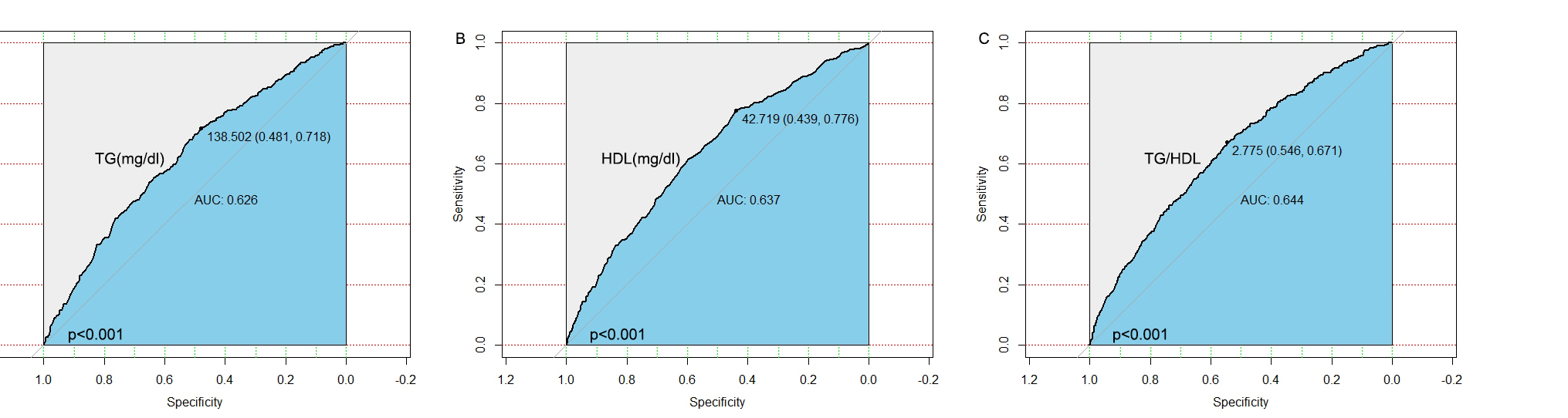

Supplement: Supplementary file 1 — Additional file 1: Supplementary Figure. Receiver operating characteristic (ROC) curve analyses of serum TG, HDL-C and TG/HDL-C ratio in sarcopenia status. [file 12944_2020_1422_MOESM1_ESM.jpg]
